# Effect of Annealing on Microstructure and Corrosion Behavior of Interstitial Free Steel

**DOI:** 10.3390/ma15010024

**Published:** 2021-12-21

**Authors:** Qiongyao He, Xiaojuan Jiang, Pengzhan Cai, Ling Zhang, Tao Sun, Xiaokui Yang, Kun Zhou, Lunwu Zhang

**Affiliations:** 1 Southwest Technology and Engineering Research Institute, Chongqing 400039, China; swsc10@yeah.net (X.Y.); 101139@cqipc.edu.cn (K.Z.); 2 College of Materials Science and Engineering, Chongqing University of Technology, Chongqing 400054, China; 3 Key Laboratory of Material Processing and Mold Technology, School of Mechanical Engineering and Automation, Chongqing Industry Polytechnic College, Chongqing 401120, China; jiangxiaojuan@cqu.edu.cn; 4 College of Materials Science and Engineering, Chongqing University, Chongqing 400044, China; pengzhancai@cqu.edu.cn (P.C.); zhangling2014@cqu.edu.cn (L.Z.); 20210901056@cqu.edu.cn (T.S.)

**Keywords:** IF steel, texture, stored energy, corrosion resistance, EBSD

## Abstract

Interstitial free steels with various grain sizes and textures were prepared by cold-rolling followed by an annealing process. The effect of grain size, crystallographic orientations and stored energy on corrosion behavior of interstitial free steel was investigated. It was found that the deformed microstructure and dislocation boundaries were consumed by recrystallizing grains during annealing. The average grain size increase ranging from 0.61 μm to 11 μm and the volume fraction of recrystallized grains was about 96% after annealing for 64 h; meanwhile, the γ fiber was the dominated recrystallized texture component. The stored energy gradually decreased due to the reduction in dislocation density by annealing. The potentiodynamic polarization and Nyquist plots show that the corrosion potential exhibits a more positive shift and depressed capacitive semicircle radius increase with rising annealing time. The 64 h annealed specimens had the biggest depressed semicircle in the Nyquist plots and the highest positive corrosion potential, which indicates the enhancement of corrosion resistance. Such an improvement of corrosion resistance is attributed to the increase in the volume fraction of the γ fiber and decrease in the stored energy.

## 1. Introduction

Interstitial free (IF) steels, as one of the most important and widely used ultralow carbon ferritic steels, are widely applied in the automotive industry due to their superior properties, such as deep drawability, fatigue resistance, high strength and weldability [1,2]. It is known that a material’s microstructure is closely related to its mechanical properties and corrosion behavior. Bulk grain refinement material can be obtained by severe plastic deformation (SPD) and grain refinement is an effective method by which to enhance mechanical strength according to the Hall–Petch relationship [3,4]. In the automotive industry, appropriate heat treatment is usually used to adjust elongation and improve formability, which enhances excellent mechanical and service properties. Furthermore, both the mechanical, service properties and corrosion resistance determine the service life of steel in specific application in the automotive industry.

The microstructure of grain refinement by SPD and grain coarsening by heat treatment affect the electrochemical corrosion behavior of metal materials because the grain boundaries of the metal material act as highly active sites and are easy to oxidize. Many studies were reported the detailed relationship between corrosion resistance and microstructure [5,6]. Ralston and Argade et al. [7,8] reported that the corrosion polarization resistance of various alloys increased with the grain refinement, which may due to the increase in the passivation ability, whereas some researchers demonstrated the contradictory results, revealing the corrosion resistance decreased with the grain refinement due to the increase in active sites in the form of the grain boundary [9]. The inconsistent reports regarding the effect of grain size on corrosion behavior may result from the fact that the influencing factors of corrosion are comprehensive factors including grain size and texture, as well as the texture changes followed by the change in the grain size during processing. Thus, it is hard to determine unilaterally influencing factors. Shahryari et al. [10] reported that the susceptibility of the surface to pitting corrosion depends strongly on the crystallographic orientation of the planes parallel to the surface in 316LVM stainless steel. Tomoyuki et al. [11] also investigated the crystallographic characteristics of grain boundaries to evaluate the corrosion susceptibility of austenitic stainless steel. Tsutsumi et al. [12] reported that the crystallographic texture and the related grain boundary density have significant effects on corrosion resistance in austenitic 316 L stainless steel fabricated by laser powder bed fusion. In addition, the effects of grain orientation spread, grain size and microstructural defect density on corrosion behaviors were extensively investigated in temper rolling [13], friction stir processing [14] and cold- and hot-rolled [15] IF steel. Nevertheless, very few studies investigated the effect of grain size and texture on corrosion behavior of cold-rolled IF steel during annealing process.

In the present study, IF steels with various grain sizes and textures were prepared by cold rolling and followed by the annealing process. The effect of grain size, texture and stored energy of crystallographic orientations on corrosion behavior under various annealing processes was investigated with open-circuit potential (OCP) and electrochemical impedance spectra (EIS) in a 3.5 wt.% NaCl solution. The relationship between stored energy, crystallographic orientations and corrosion resistance were discussed.

## 2. Experimental

The starting material was ~77.8% cold-rolled (CR) commercial IF steel with a chemical composition of 0.0012 wt% C-0.005 wt% Si-0.1 wt% Mn-0.061 wt% Ti. The annealed specimens with dimensions of 10 mm × 8 mm × 1 mm were cut from starting materials. Six specimens were annealed, respectively, at 600 °C for 2 h, 4 h, 8 h, 16 h, 32 h and 64 h to gain different volume fractions of recrystallized microstructures. The microstructures of the as-rolled and annealed samples were characterized using electron backscatter diffraction (EBSD) on the longitudinal plane, i.e., the rolling direction (RD) and normal direction (ND) section. Step sizes of 0.1 μm were used for EBSD analysis of the as-rolled and annealed samples, while a step size of 0.1–0.5 μm was used for characterizing partially recrystallized samples.

The recrystallized grains in the annealed samples were automatically detected from the EBSD data by the method described in ref [16]. The recrystallized grains were identified with an equivalent circular diameter (ECD) larger than 1.5 μm, at least partly surrounded by high angle boundaries with misorientation of more than 15° and internal misorientation of less than 1°. The volume fraction of recrystallized grains was measured for each annealing period as the area fraction of recrystallized grains in the measured maps. Fractions of different texture components were calculated with 15° deviation from chosen ideal orientations. The stored energy of boundaries per unit volume was calculated from the EBSD data using the method described in ref [17]. The specific boundary energy of low angle boundaries was calculated from the Read–Shockley equation [18,19]. The specific boundary energy of high angle boundaries was taken as 0.617 J/m^2^ [20].

The electrochemical measurements were carried out in a CH630D electrochemical workstation at room temperature in 3.5 wt.% NaCl solution. A conventional three-electrode system with a saturated calomel electrode as reference electrode, a platinum sheet as the counter electrode, and an IF steel sample as the working electrode were used. Before electrochemical measurements, the open-circuit potential (OCP) value was measured in the test solution for approximately 30 min to form a stable passive film. Electrochemical impedance spectra (EIS) tests were carried out at the stabilized OCP within a frequency range of 100 kHz to 10 mHz and a sinusoidal perturbation with 10 mV amplitude. The potentiodynamic polarization curves were constructed by scanning varying from −0.7 V_SCE_ to −0.4 V_SCE_ at a sweep rate of 1 mV/s. Four samples were tested in the parallel experiments. All the data were analyzed by IviumSoft. Tafel slope fitting was employed to obtain the corrosion current density (I_corr_).

## 3. Results and Discussion

### 3.1. Effects of Annealing on Microstructure and Texture

**Figure 1:**
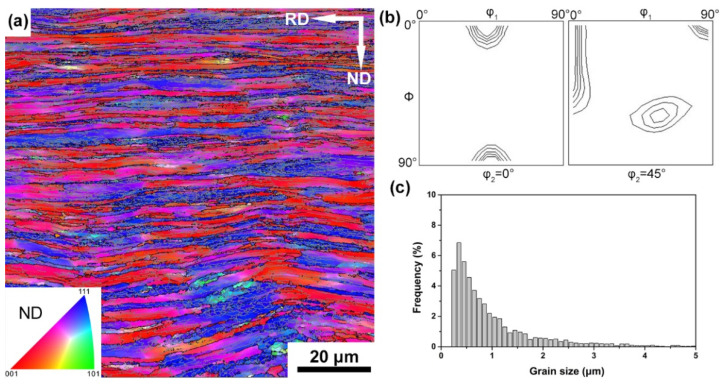
EBSD IPF grain orientation map (**a**), ODF sections representing the texture map (**b**) and the grain size distribution map (**c**) in as-rolled IF samples. Different colors in the maps correspond to different crystallographic directions along the ND as shown in the inset in (**a**). Contour layers of ODF: 1, 3, 5, 10, 20, 30, 40× random.

The microstructure of the as-rolled sample on the longitudinal plane, as shown in Figure 1a, is typical of body-centered cubic (bcc) steels deformed to a similar strain, which were composed of a-few-micrometer-thick bands with different crystallographic orientations [21,22]. The bands were elongated along the RD and separated by HABs (high angle grain boundaries) with a misorientation of more than 15°. Inside each band, the microstructure was further subdivided by deformation-induced boundaries with mostly LABs (low angle grain boundaries) and occasionally HABs. The average boundary spacing that measured by the line intercept method along ND is ~0.61 μm (Figure 1c). After rolling, a typical rolling texture in BCC metals with components commonly called γ-fiber (〈111〉//ND), α-fiber (〈110〉//RD) and a {100}〈110〉 components was developed [23], as shown by the ODF in Figure 1b. The α-fiber appears in red and the γ-fiber is presented in blue in Figure 1a, forming bands in the as-rolled microstructure.

**Figure 2:**
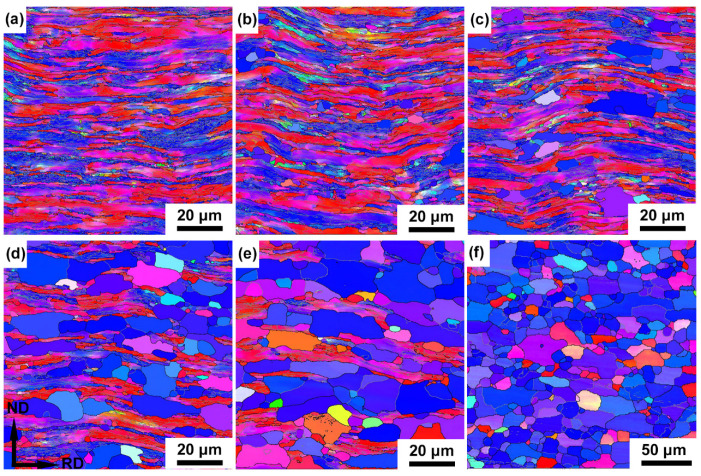
Orientation maps of partially recrystallized microstructures of the cold-rolled IF steel annealed at 600 °C for (**a**) 2 h, (**b**) 4 h, (**c**) 8 h, (**d**) 16 h, (**e**) 32 h and (**f**) 64 h.

**Figure 3:**
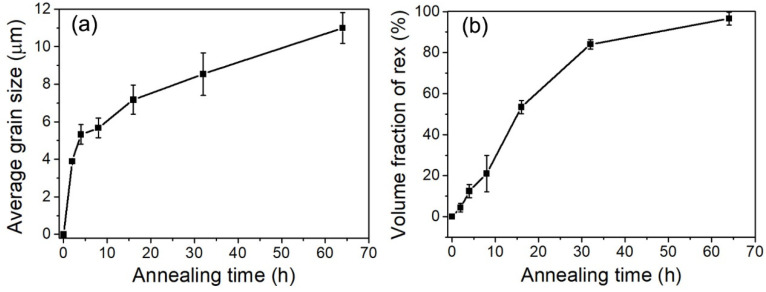
Changes in the average Rex grain size (**a**) and volume fraction of the Rex grain (**b**) of the cold-rolled IF steel during annealing at 600 °C for varying time periods.

Figure 2 and Figure 3 show the microstructure evolution of cold-rolled IF after annealing at 600 °C for a varying time. The microstructure coarsens to ~790 nm from ~610 nm of original IF steel after annealing for 2 h, and the extended bands bulged, accompanied by the decrease in the aspect ratio of subgrains (Figure 2a). Continued bulging between 2 h and 4 h resulted in the onset of recrystallization (Figure 2b). Nucleation predominantly occurred at a highly deformed region, i.e., shear bands and HABs. The Rex grains are more or less equiaxed with an average grain size of ~5.3 μm (Figure 3a). After 8 h of annealing, the volume fraction of recrystallized grains was 21% and the average grain size increased to ~5.7 μm (Figure 2c and Figure 3a,b). The structures in the deformed matrix continue to coarsen with further annealing. The volume fraction of the Rex grains exceeds 53% after annealing for 16 h (Figure 2d and Figure 3b), and the unRex matrix gradually becomes surrounded by the Rex grains. The impingement between two unRex bands leads to a string of Rex grains parallel to RD, whereas grains preferentially nucleating within shear bands that tend to align at ±30° to RD. After annealing for 64 h, the deformed elongated substructures were consumed by recrystallized grains and the microstructure was almost fully recrystallized with a recrystallized volume fraction of ~96% and an average grain size of ~11 μm (Figure 2d and Figure 3b).

Comparing the different crystallographic orientations of γ-fiber (〈111〉//ND), α-fiber (〈110〉//RD) and {100}〈110〉 component, recrystallized grains more frequently are γ-fiber (〈111〉//ND, blue in Figure 2), in which they are further increased and almost occupy the whole space with increasing annealing time. Compared to the average grain size of specimens after annealing from 8 h to 64 h, the coarsening rate of recrystallized grains is more pronounced after annealing for 8 h. This difference in the recrystallized grains’ coarsening rate is likely to involve the evolution of high angle and low angle grain boundaries during the annealing process [24]. Meanwhile, the changes of fraction of high angle and low angle grain boundaries are closely related to the recrystallization process.

**Figure 4:**
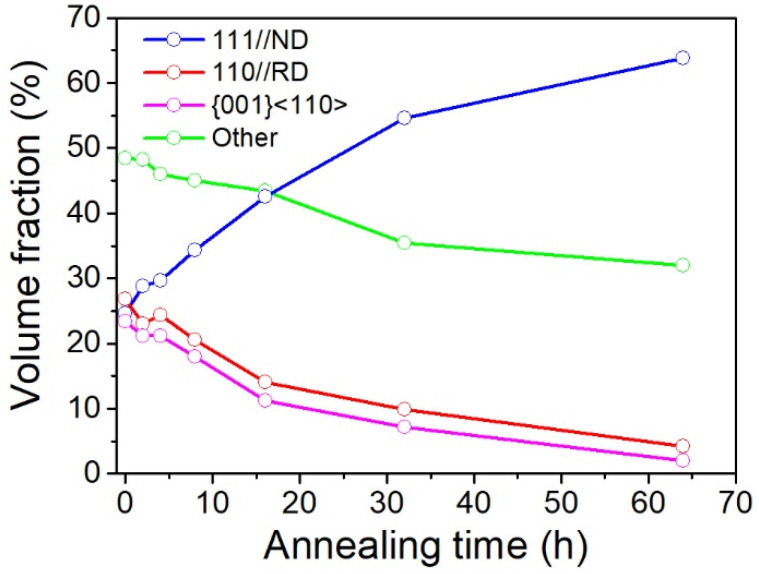
Changes in the volume fraction of Rex grains in the cold-rolled IF steel during annealing at 600 °C for varying time periods. The Rex grain is presented separately for grains of 〈111〉 //ND, 〈110〉 //RD, {001}〈110〉 and other texture components.

Changes in the volume fraction of Rex grains in the cold-rolled IF steel during annealing at 600 °C for varying time periods are shown in Figure 4 and Appendix A. The cold-rolled original specimen exhibits a fairly random but with a little weak γ-fiber texture, α-fiber and {001}〈110〉 component. The volume fractions of the three texture components are ~25%, 27% and 24%, respectively. During annealing, the α-fiber and {001}〈110〉 component is unstable, and its volume fraction was gradually decreased, whereas the intensity of γ-fiber turns to be the dominated texture components. After recrystallization, the γ fiber texture occupies 64% of the area. With improvement of the γ fiber texture component and decrease in the α-fiber component, the corrosion resistance was enhanced.

The strong correlation of corrosion resistance and the γ fiber texture indicates that the formation of the γ fiber texture leads to enhancement of the corrosion properties. On the other hand, previous studies have investigated the effect of texture on corrosion. Wang and Lv [25,26] reported that the significant decrease in (110) plane of cold-rolled samples reduces their corrosion resistance in pure iron. Meanwhile, serve deformation increased the 〈111〉//ND texture component and thus increased their corrosion resistance. Similar conclusions were also reported by Masoumi et al. [27] in X70 pipeline steel, which highest hydrogen-induced cracking resistance was obtained in sample rolled isothermally at 850 °C, due to the high proportion of grains oriented with 〈111〉//ND. It can be concluded, therefore, that the grains with the γ fiber component’s crystallographic orientation can has a significant impact on the corrosion resistance in steel.

### 3.2. Corrosion Behavior of the Various Annealed IF Steel

**Figure 5:**
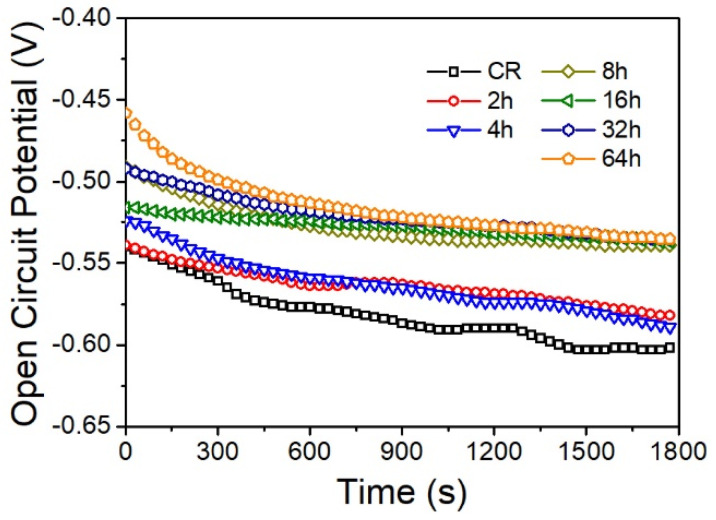
OCP curves of various annealed IF steel specimens.

Figure 5 shows the relationship between OCP and test time of various annealed specimens in 3.5 wt.% NaCl solution at room temperature. With increasing exposure time of the tested specimens in electrochemical solution, all the curves move gradually towards to more negative values, indicating that the passive films formed in open air are destroyed on the electrode surfaces under the influence of electrochemical solution. With the rise in immersed time, the negative tendency of corrosion potential (E_corr_) gradually increases and finally the reaches a quasi-steady state after being immersed for 1500 s. Among all the curves, the variation of the E_corr_ is negligible (less than 0.02 mv/s) after E_corr_ achieves a quasi-steady state. All the curves during the whole immersion period show a slight difference. For example, the E_corr_ value of the 64 h annealed specimen is about −0.53 V_SCE_, whereas that of the deformed unprocessed specimen is about −0.59 V_SCE_. Moreover, the E_corr_ values of the 2h, 8h and 32h annealed specimen, between the two most positive and the most negative specimens, are −0.54 V_SCE_, −0.54 V_SCE_ and −0.58 V_SCE_, respectively. As a result, the 64 h annealed specimen has the best corrosion resistance due to it having the most positive value (−0.53 V_SCE_).

**Figure 6:**
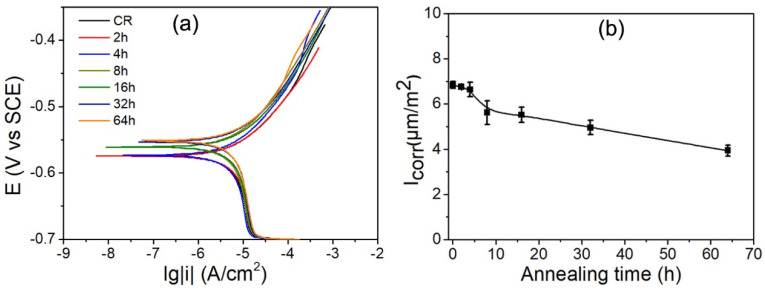
Potentiodynamic polarization curves (**a**) and calculated corrosion current density, I_corr_, (**b**) of various annealed IF steel specimens.

The potentiodynamic polarization, including cathodic branches and anodic branches, and calculated corrosion current density, Icorr, were used to investigate the process of cathodic hydrogen evolution and anodic dissolution of tested specimens, respectively, as shown in Figure 6. The anodic and cathodic branches of polarization curves were not symmetrical. All the specimens including with and without annealing specimens show similar passivation behaviors in anodic branches. The electrochemical data of the free corrosion potential (E_corr_) and corrosion current density (I_corr_) were obtained from the polarization curves by Tafel extrapolation. The E_corr_ is −0.57 V for the deformed specimen. With increasing annealing time, the corrosion potential shows a more positive shift. The E_corr_ of 64 h annealed specimen are −0.55 V, which is more positive (−0.57 V) than the deformed specimen. The deformed specimen has highest corrosion rate, and the 64 h specimen has the lowest corrosion rate. It is indicated that corrosion tendency and corrosion rates decline with increasing annealing time.

**Figure 7:**
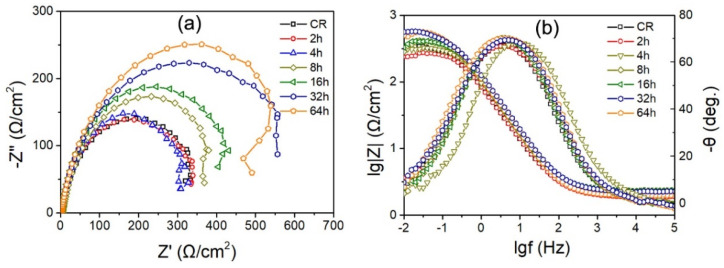
Nyquist plots (**a**) and Bode plots (**b**) of various annealed IF steel specimens.

The electrochemical corrosion behavior of annealed IF steel specimens was further investigated by electrochemical impedance spectroscopy in the form of Nyquist and Bode plots, as shown in Figure 7a,b, respectively. It was observed that all curves exhibit a similar depressed capacitive semicircle with various semicircle radii (Figure 7a). The radius of the capacitive semicircle is associated with the stability and compactness of passive film. The larger the radius of capacitive semicircle, the higher the passive film stability for the IF steel specimens. The cold-rolled IF steel specimen has the smallest depressed semicircle, but the 64 h annealed specimen has the biggest depressed semicircle, illustrating that it has best corrosion resistance. On the other hand, with increasing frequency, the value of |Z| decreases for all the specimens in the entire frequency. At moderate frequencies, the Bode angle value is highest and the value of log|Z| is linear with log(f), and this may due to the formation of flat and compact passive films. At high and low frequencies, the value of log|Z| varies gently with frequency, which may result from the comprehensive effect of resistive and capacitive factors. The 64 h annealed specimen has maximal value of |Z| and the highest angle among all the specimens, as shown in Figure 7b, indicating that it has the highest corrosion resistance.

### 3.3. Effects of Texture and Stored Energy on Corrosion Behaviors

The stored energy was used to investigate the corrosion behavior of annealed specimens. According to the Read–Shockley equation [18], it is expressed as:

(1) $$\gamma \left(\theta \right)=\gamma_{m}\left(\theta /\theta_{m}\right)\left[1-ln\left(\theta /\theta_{m}\right)\right]:\theta \langle =\theta_{m}\gamma \left(\theta \right)=\gamma_{m}:\theta \rangle \theta_{m}$$

where $\gamma_{m}$ is the energy per unit area of a high-angle boundary, *θ* the boundary misorientation, and $\theta_{m}$ the misorientation angle above which the energy per unit area is independent of the misorientation angle. As shown in the Read–Shockley equation, the γ(θ) is associate with the tilt–twist character of the boundary. The stored-energy-per-unit volume can be obtained by multiplying the energy-per-unit boundary area (γ(θ)) with the area-per-unit volume ($S_{V}$) for the boundary, based on dislocation boundary of a given misorientation angle. Then, the total stored energy can be described as:

(2) $$E_{s}=S_{V}\gamma \left(\theta \right)+E\left(\rho_{0}\right)$$

where $E\left(\rho_{0}\right)$ is stored energy of any individual dislocations which are located in dislocation boundaries. In the calculation, $E\left(\rho_{0}\right)$ is normally neglected, since the contribution of $E\left(\rho_{0}\right)$ is small for medium and high-stacking fault-energy materials [17].

**Figure 8:**
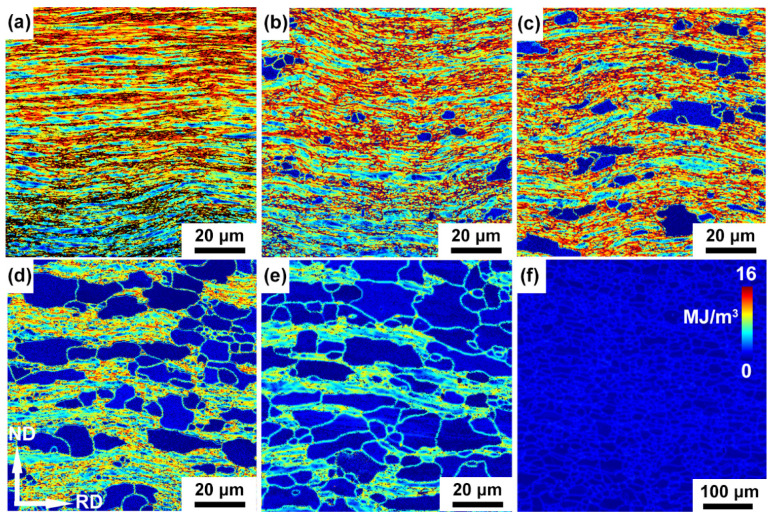
Stored energy maps of the cold-rolled IF steel annealed at 600 °C for (**a**) 0 h, (**b**) 4 h, (**c**) 8 h, (**d**) 16 h, (**e**) 32 h and (**f**) 64 h.

The stored energy of cold-rolled and annealed IF steel specimens are shown in Figure 8. The deformed IF steel has highest stored energy and the grains with high stored energy homogeneously are distributed in the whole specimen. During the cold rolling, deformation-induced dislocations were largely introduced, meanwhile the stored energy increases with rise in the dislocation density. After annealing, the stored energy decreases and the stored energy gradually reduced to a minimum with increasing annealing time up to 64 h. This may be due to the decrease in the dislocations density when recrystallized grains with low dislocation density replace the deformed grains containing large amounts of dislocation density during the annealing process (Figure 8b–f).

Previous reports have discussed the dislocations introduced by deformation enhance stored energies in iron and steel, leading to degradation of corrosion resistance [28,29]. Moreover, the dislocation density and stored energy determine the degree and rate of the erosion [30]. The higher stored the energy, the higher the corrosion tendency. Then the deformed specimens with highest stored energy have quick corrosion rates. With the decrease in the stored energy, the ability of corrosion resistance improves. Therefore, 64 h annealed specimens with the lowest stored energy have the best corrosion resistance.

**Figure 9:**
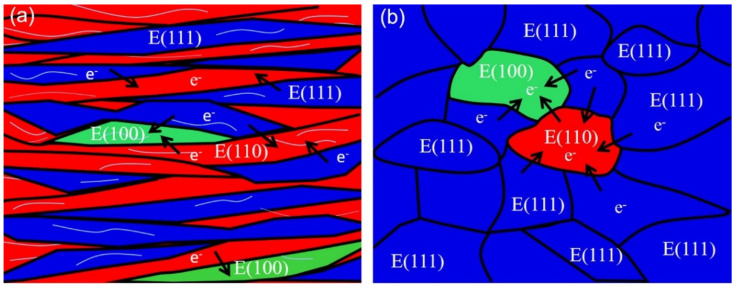
Schematic diagram of the effect of texture on the corrosion behavior of cold-rolled IF steel before and after annealing.

The schematic diagram of the effect of texture and dislocation on the corrosion behavior of cold-rolled IF steel before and after annealing is shown in Figure 9. On the one hand, a large number of LABs (thin grey line in Figure 9a) were formed by dislocation rearrangement and accumulation during deformation. Meanwhile, the dislocation is quickly multiplied by cold rolling. Then, stored energy improves due to plentiful dislocation density in cold-rolled IF steel, leading to weak corrosion resistance. After annealing, most of the LABs and the substructure disappear due to grain nucleation and growth. Then, the stored energy decreases during annealing.

On the other hand, the various grain orientations (〈111〉//ND, 〈110〉//RD and a {100}〈110〉 component) occasionally distribute in IF specimens during cold rolling (Figure 9a,b). It was reported that that the difference in corrosion tendency and rate was found between 〈111〉//ND and 〈110〉//RD in iron bi-crystal and polycrystalline samples [31]. Then, the corrosion potential of adjacent grains becomes different due to the different orientations of the neighboring grains. As a result, the different orientations in neighboring grains result in a quick corrosion rate. The α-fiber and {001}〈110〉 components are unstable during annealing. After annealing, the volume fraction of the α-fiber and {001}〈110〉 component were gradually decreased, whereas the γ-fiber, dominating texture components, occupies most of the texture volume fraction (Figure 9b). The plentiful γ-fiber reduces the difference in adjacent grains’ orientations, leading to the small difference in the corrosion potential and diminished corrosion rate. To sum up, the corrosion resistance enhances due to the reduction in stored energy and preferred orientation growth of grains.

## 4. Conclusions

IF steels with various grain size and texture were prepared by cold-rolling and this was followed by an annealing process. The effect of grain size, texture and stored energy of crystallographic orientations on corrosion behavior was investigated. The major findings in the current study are as follows:

(1) Grains grow and coarsen from about 0.61 μm to about 11 μm and deformed elongated substructures are consumed by recrystallized grains, and the volume fraction of recrystallized was about 96% after annealing for 64 h.
(2) The volume fractions of the γ-fiber texture, α-fiber and {001}〈110〉 component are about 25%, 27% and 24%. The α-fiber and {001}〈110〉 component decrease, and the intensity of γ-fiber increases after annealing.
(3) The stored energy gradually reduces as the annealing time increases, because the recrystallized grains replace the deformed grains during the annealing process.
(4) The value of |Z| and the semicircle radius of depressed capacitive semicircle increase with increasing annealing time, and the 64 h annealed specimen has the biggest depressed semicircle and maximal value of |Z|, indicating that corrosion tendency and corrosion rates decline with increasing annealing time under the effect of the texture and stored energy.

## Figures and Tables

**Figure A1:**
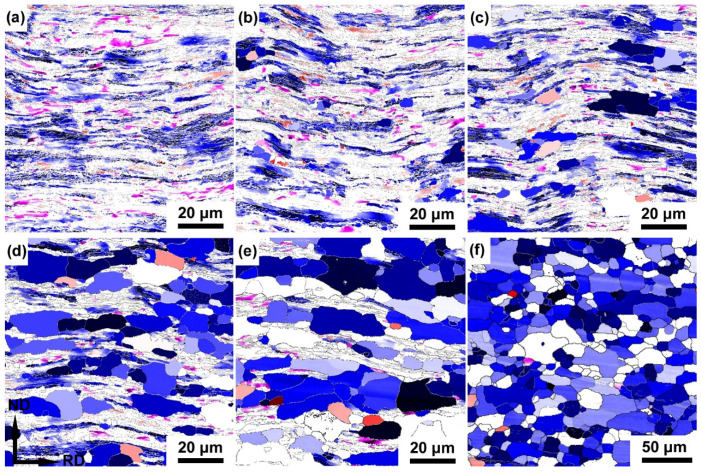
Texture components and their corresponding Euler angles of the cold rolled IF steel annealed at 600 °C for (**a**) 2h, (**b**) 4h, (**c**) 8h, (**d**) 16h, (**e**) 32h and (**f**) 64h. The texture component of 〈111〉 //ND is presented by gradient blue 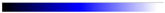. The texture component of 〈110〉 //RD is presented by gradient red 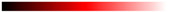. The texture component of {001}〈110〉 is presented by gradient fushcia 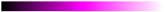.

## Data Availability

This study did not report any data.
